# Relationship Between Final Visual Acuity and Optical Coherence Tomography Findings in Patients with Diabetic Macular Edema Undergoing Anti-VEGF Therapy

**DOI:** 10.4274/tjo.galenos.2019.91962

**Published:** 2020-06-27

**Authors:** Seher Eraslan, Özlem Yıldırım, Özer Dursun, Erdem Dinç, Gülhan Orekici Temel

**Affiliations:** 1 Nevşehir State Hospital, Clinic of Ophthalmology, Nevşehir, Turkey; 2 Mersin University Faculty of Medicine, Department of Ophthalmology, Mersin, Turkey; 3 Mersin University Faculty of Medicine, Department of Biostatistics, Mersin, Turkey

**Keywords:** Diabetic macular edema, optical coherence tomography, anti-VEGF treatment

## Abstract

**Objectives:**

To identify the prevalence of findings in optical coherence tomography (OCT) sections before intravitreal anti-VEGF treatment in patients with diabetic macular edema (DME), and to evaluate the relationship between these findings and final visual acuity and number of injections.

**Materials and Methods:**

This retrospective study included 296 eyes of 191 patients (104 male, 87 female) who started intravitreal ranibizumab treatment after being diagnosed with DME in the retina unit between January 2013 and April 2017 were included the study. Spectral domain OCT findings at the time of presentation such as presence of serous macular detachment (SD), vitreomacular traction (VMT), and epiretinal membrane (ERM) were recorded. In addition, the regularity of the ellipsoid zone (EZ) and inner retinal layers was also studied.

**Results:**

The mean central retinal thickness measured in SD-OCT was 449±81 μm before treatment and 350±96 μm after treatment (p<0.001). SD was detected in 155 eyes (52.4%), ERM in 67 eyes (22.6%), and VMT in 9 eyes (3%). Thirty eyes (10.1%) had disorganization of the retinal inner layers (DRIL) and 54 eyes (18.2%) had EZ deterioration. The presence of ERM, EZ irregularity, and DRIL were associated with significantly lower final visual acuity (p<0.0001), while there was no relationship between pre-treatment SD and final visual acuity (p=0.11). Injection number was higher in eyes with SD and ERM compared to those without, but this difference was statistically significant only in the presence of SD (p=0.01 and p=0.59, respectively). There was no difference in injection number according to EZ irregularity or presence of DRIL.

**Conclusion:**

The coexistence of SD with DME was associated with increased need for treatment but not with final visual acuity. EZ irregularities, DRIL, and ERM are findings that negatively affect visual acuity.

## Introduction

Diabetic retinopathy (DR) is an important complication of diabetes and is closely associated with disease duration. DR is among the leading causes of acquired vision loss in adults worldwide. Diabetic macular edema (DME) is a serious and characteristic complication of DM-related maculopathy and is the most common cause of vision loss in these patients.^1,2^ DME can emerge at any stage of DR, and its prevalence is expected to increase with that of diabetes, as is the case with DR. The global prevalence was 8.3% in 2013 and is projected to increased to 10.1% by 2035.^3^ Two different studies conducted in Turkey reported the prevalence of DME as 14.2% and 15.3%.^4,5^

Optical coherence tomography (OCT) is a noninvasive, noncontact imaging method that allows in vivo, quantitative imaging of the human retina with high-resolution sections. It is the only method that provides cross-sectional images of the anatomic and topographic structure and pathologies of the retinal layers.^5,6^ OCT has become an important diagnostic tool due to the information it provides about vitreoretinal relationships and the internal structure of the retina in the assessment and monitoring of DR. The use of OCT has not only made it possible to objectively evaluate DME, but also to make new descriptions such as serous macular detachment (SD). In addition, OCT has advanced our understanding of the importance of vitreoretinal interface pathologies in the pathogenesis of DME and their impact on treatment response. Thanks to newly described OCT findings, personalized information can be obtained about disease severity, treatment response, and prognosis. In addition to all of these, in the current era of anti-VEGF therapy, drug effectiveness is assessed using OCT, which has further increased the importance of OCT in the treatment monitoring of macular edema.

The present study aims to evaluate the relationship between pre-treatment OCT findings and final visual acuity and number of injections in patients who presented with complaints of low visual acuity due to DME and underwent anti-VEGF therapy.

## Materials and Methods

The study was conducted after obtaining approval from the Mersin University Clinical Research Ethics Committee (decision number 2017/284). The study included 296 eyes of 191 patients (104 men and 87 women) who presented to the ophthalmology department of the Mersin University Faculty of Medicine with complaints of decreased vision between January 2013 and April 2017, were diagnosed as having DME in the retina unit, and were started on intravitreal ranibizumab therapy. Patients whose records included detailed medical history, complete examination findings, and OCT sections suitable for evaluation, had no history of previous intravitreal therapy or retinal surgery, and attended regular follow-up appointments were included in the study. Patients whose records were incomplete, whose OCT images could not be obtained due to media opacity, who had a history of previous intravitreal therapy or retinal surgery, or did not attend regular follow-up appointments were excluded from the study. The visual acuity, examination findings, and OCT data of all patients who met the inclusion criteria were screened and recorded.

OCT images (Cirrus 4000 HD-OCT, Zeiss Meditec) of all patients were evaluated in terms of the presence of vitreomacular traction (VMT), epiretinal membrane (ERM), SD, disorganization of the retinal inner layers (DRIL), and integrity of the ellipsoid zone (EZ). The relationship between OCT findings and the number of injections and final visual acuity were statistically evaluated.

### Statistical Analysis

Conformity of the data to normal distribution was evaluated using Shapiro-Wilk test. Descriptive statistics were expressed as mean and standard deviation for normally distributed data and as median and percentage values for nonnormally distributed data. Categorical parameters were expressed as numbers and percentages. Differences between two groups were evaluated using Student’s t-test for parameters that showed normal distribution and Mann-Whitney U test for parameters that did not. Kruskal-Wallis test was used to evaluate differences between more than two groups. A paired-samples t-test was used to analyze pre- to post-treatment changes, chi-square test was used to analyze relationships between categorical parameters, and correlation analysis was used to evaluate relationships between continuous parameters. The data were analyzed using SPSS 11.5 package software. P<0.05 was set as the threshold for statistical significance.

## Results

**Figure 1:**
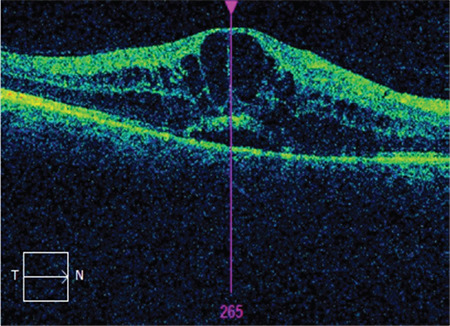
Optical coherence tomography shows serous macular detachment in the right eye of a patient with cystoid macular edema

**Figure 2:**
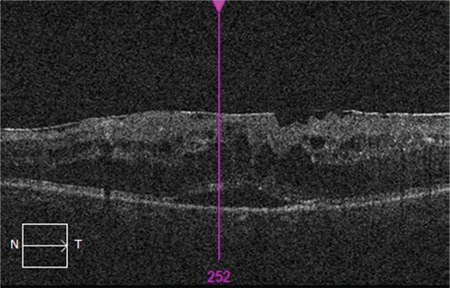
Optical coherence tomography demonstrates coexistence of epiretinal membrane and serous macular edema in a patient with diabetic macular edema

**Figure 3:**
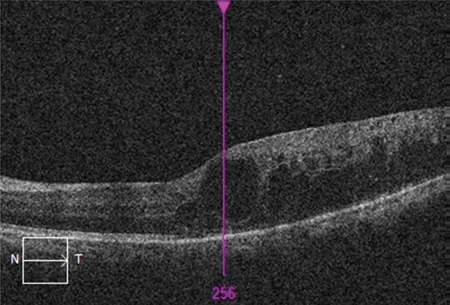
OCT reveals DRIL and irregular ellipsoid zone in the left eye of a patient with cystoid macular edema. Although the macular edema completely regressed over a 36-month follow-up period, there was no improvement in visual acuity OCT: Optical coherence tomography, DRIL: Disorganization of retinal inner layers

**Figure 4:**
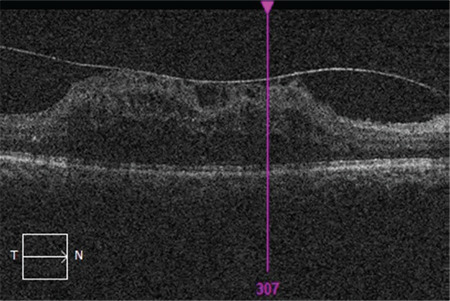
Extensive vitreomacular traction is observed in a patient with diabetic macular edema

The mean age of the patients included in the study was 60.96±8.58 years and the mean disease duration was 15.49±7.78 years. The patients’ mean follow-up time was 19.61±9.31 months and they received a mean of 5.92±2.77 injections during this time. Best corrected visual acuity was 0.3±0.22 before intravitreal injection and 0.36±0.26 after injection (p<0.001). Similarly, central retinal thickness was 449±81 µm before treatment and 350±96 µm after treatment (p<0.001). The most common OCT finding in the eyes included in the study was SD, which was detected in 52.4% of the eyes (Figure 1). Other than these findings, ERM was present in 22.6% of the eyes (Figure 2), DRIL in 10.1% (Figure 3), and VMT in 3% (Figure 4). In addition, EZ irregularity was observed in 18.2% of the eyes included in the study (Table 1).

Although eyes with SD and ERM received more injections compared to eyes without, this difference was only statistically significant for eyes with SD (p=0.01 and p=0.59, respectively). In contrast, EZ irregularity and DRIL were not significantly associated with number of injections (p=0.84 and p=0.4, respectively) (Table 2). When the relationship between OCT findings and final visual acuity was evaluated, there was no statistical relationship between final visual acuity and presence of SD, whereas presence of ERM was associated with significantly lower final visual acuity (p=0.11 and p<0.0001, respectively). EZ irregularity and DRIL were also associated with significantly lower final visual acuity (p<0.0001 for both) (Table 3).

## Discussion

With the widespread use of OCT in the diagnosis and monitoring of retinal diseases, we have gained a better understanding of the importance of the vitreomacular interface. In addition, new pathologies have been identified in relation to retinal diseases and researchers have started to investigate the relationship between these pathologies and visual outcomes. Similar developments have occurred for DME patients, as OCT has enabled identification of various pathologies such as the presence SD, DRIL, EZ irregularity, and hyperreflective dots in these patients and these findings have started to shed light on both the pathogenesis and prognosis of the disease.

In OCT studies of patients with DME, the prevalence of SD has been found to range from 11.4% to 51.9%.^8,9,10,11,12^ In the present study, the most common OCT finding observed in patients was SD, which was present in approximately half of the patients. This result is consistent with the literature, as the results of more recent studies demonstrate a higher prevalence of SD compared to earlier studies. This phenomenon parallels advances in OCT technology, which have made it possible to obtain higher quality images and more clearly visualize pathologies that were previously overlooked. The mechanism underlying the development of SD is not fully known, but a study by Turgut et al.^13^ suggested that there may be a relationship between serum HbA1_c_ levels and the development of SD and that SD may occur due to impaired retinal pigment epithelium functions in patients with poor metabolic control.

Ozdemir et al.^14^ first showed that SD observed in patients with DME may regress after intravitreal triamcinolone treatment and that an increase in visual acuity may be achieved. In another study, Maalej et al.^15^ suggested that the presence of SD was associated with low visual acuity. However, Murakami et al.^16^ asserted that the presence of SD in DME patients was not associated with low visual acuity. Seo et al.^17^ proposed a different view, stating that visual prognosis is related to the initial deterioration in the photoreceptor layer and that this occurs more commonly in the presence of SD. All these studies show that the impact of SD on visual prognosis in patients with DME is controversial. In the present study, the presence of SD was not associated with visual outcomes. However, the more interesting result is that DME patients with SD received more injections compared to patients without SD. In a study conducted by Koytak et al.^18^ evaluating patients treated with intravitreal bevacizumab, it was found that the decrease in central retinal thickness was greater in the cystoid macular edema group and SD group, but that there was no effect on visual prognosis. Kim et al.^19^ emphasized that although eyes with SD responded better to intravitreal bevacizumab injections, they also required the administration of repeated doses. The high number of injections received by patients with SD may be due to the fact that these patients respond well to treatment but need repeated injections. In addition, the presence of SD may also be associated with edema severity. This is supported by the results obtained in the present study.

In OCT studies of DME patients, the prevalence of ERM has been found to vary between 10.92% and 34.5%.^10,20,21,22^ The prevalence of ERM in the DME patients in the present study was 22.6%, consistent with the literature. However, these patients should be monitored long-term for the development of ERM. Kulikov et al.^23^ compared the visual acuity outcomes of DME patients with and without ERM and determined that the patients with ERM had poorer visual acuity and showed a more limited response to treatment compared to those without ERM. Lai et al.^24^ also stated that treatment response was limited in DME patients with ERM but that visual acuity at 3 months was not affected. The main limitation of their study was the short follow-up period; in a study by Wong et al.^25^ with longer follow-up period, the presence of ERM was shown to adversely affect visual acuity. The mean follow-up time in the present study was longer than in all of these three studies, and it was found that the presence of ERM was associated with worse final visual acuity but did not affect the number of injections. This result is not surprising, as it is clear that the presence of ERM will have a negative impact on the anatomical structure of that region.

Another new concept related to patients with DME that has emerged in recent years due to developments in OCT technology is DRIL. DRIL is defined as the inability to distinguish any of the borders separating the retinal inner layers (ganglion cell layer-inner plexiform layer complex, inner nuclear layer, and outer plexiform layer). DRIL was detected in 10.1% of the eyes included in the present study. Studies show that the presence of extensive DRIL is associated with poor visual outcomes and that this is associated with capillary perfusion disorder.^25,26,27,28,29^ Nicholson et al.^27^ stated that the presence of DRIL was an indicator of macular capillary nonperfusion. According to their study, DRIL had 84.4% sensitivity and 100% specificity in the detection of macular capillary non-perfusion. Considering that fundus fluorescein angiography is an invasive method, the evaluation of DRIL may enable macular ischemia to be detected without performing an invasive procedure. DRIL may regress over time and this is associated with improvements in vision. In other words, the regression of DRIL actually indicates anatomical recovery and a return to a more normal morphology.^27^ DRIL is a good indicator of whether an eye’s vision will increase or decrease with treatment, and thus is a good guide for predicting visual prognosis. The findings obtained from the present study are also consistent with the literature, and the presence of DRIL has been shown to adversely affect visual prognosis. If DRIL is considered an anatomic defect, this anatomic defect will inevitably affect vision. Interestingly though, a significant relationship was not established between the presence of DRIL and number of injections.

Another concept emphasized in DME patients is EZ integrity. This layer, previously referred to as the IS/OS band, was believed to be associated with the photoreceptor inner segments, and the second hyperreflective band seen on OCT was named the EZ as a result of the consensus reached in international terminology.^30^ Both the EZ and the external limiting membrane are an important indicators of photoreceptor integrity and are used as indicators in predicting the visual prognosis of patients with DME. In a study by Iacono et al.,^31^ the integrity of both the EZ and the ELM were shown to be closely related with final visual acuity. A similar result was also reported by Mori et al.^32^ In addition, EZ integrity has been shown to be a good indicator of treatment response.^33,34^ EZ irregularity was observed in 18.2% of the eyes in the present study, and the visual acuity outcomes of these patients were significantly poorer compared to patients without EZ irregularity. However, a significant relationship was not observed between EZ irregularity and injection number. This result is consistent with studies in the literature. Similar to the presence of DRIL, anatomic disruption in the central macula adversely affects visual acuity and is reflected in the visual acuity achieved after treatment.

## Conclusion

In the present study, it was found that OCT findings obtained from patients with DME may be related to injection number and to visual prognosis in particular. In light of these findings, informing patients before the treatment about visual prognosis will be greatly beneficial for both the physician and the patient and will prevent unexpected surprises. Conducting this and similar studies with new devices that can yield more detailed images will provide better opportunities both to identify new pathologies and to evaluate the effects of these pathologies on visual outcomes compared to currently available technology.

## Figures and Tables

**Table 1:**
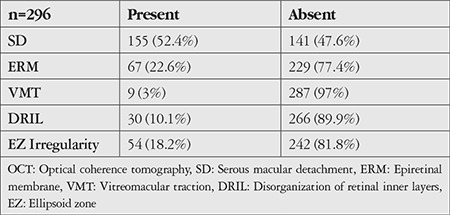
Distribution of pre-treatment OCT findings

**Table 2:**
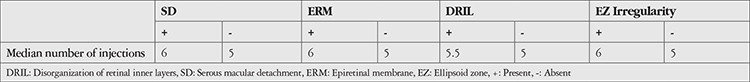
Number of injections in patients with and without serous macular detachment, epiretinal membrane, DRIL, and irregular ellipzoid zone

**Table 3:**
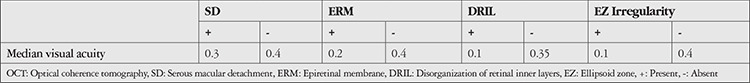
Relationship between OCT findings and final visual acuity

## References

[1] Chew EY, Klein ML, Ferris FL, Remaley NA, Murphy RP, Chantry K, Hoogwerf BJ, Mİller D (1996). Association of elevated serum lipid levels with retinal hard exudate in diabetic retinopathy. Early Treatment Diabetic Retinopathy Study (ETDRS) report 22. Arch Ophthalmol..

[2] Klein R, Klein BE, Moss SE, Cruickshanks KJ (1995). The Wisconsin Epidemiologic Study of Diabetic Retinopathy. XV. The long-term incidence of macular edema. Ophthalmology..

[3] Sultan MB, Zhou D, Loftus J, Dombi T, Ice KS, Macugen 1013 Study Group (2011). A phase 2/3, multicenter, randomized, double-masked, 2-year trial of pegaptanib sodium for the treatment of diabetic macular edema. Ophthalmology..

[4] Taş A, Bayraktar MZ, Erdem Ü, Sobacı G (2005). Prevalence and risk factors for diabetic retinopathy in Turkey. Gulhane Med J..

[5] Acan D, Calan M, Er D, Arkan T, Kocak N, Bayraktar F, Kaynak S (2018). The prevalence and systemic risk factors of diabetic macular edema: a cross-sectional study from Turkey. BMC Ophthalmol..

[6] Kim BY, Smith SD, Kaiser PK (2006). Optical coherence tomographic patterns of diabetic macular edema. Am J Ophthalmol..

[7] Knudsen LL (2007). Identification of diabetic macular edema using retinal thickness measurments. Acta Ophthalmogica Scandinavica..

[8] Otani T, Kishi S, Maruyama Y (1999). Patterns of diabetic macular edema with optical coherence tomography. Am J Ophthalmol..

[9] Koleva-Georgieva D, Sivkova N (2009). Assessment of serous macular detachment in eyes with diabetic macular edema by use of spectral-domain optical coherence tomography. Graefes Arch Clin Exp Ophthalmol..

[10] Ozdemir H, Karacorlu M, Karacorlu S (2005). Serous macular detachment in diabetic cystoid macular oedema. Acta Ophthalmol Scand..

[11] Yaya O, Taş İ (2015). The Frequency of Serous Macular Detachment in Diabetic Macular Edema. Turk J Ophthalmol..

[12] Shereef H, Comyn O, Sivaprasad S, Hykin P, Cheung G, Narendran N, Yang YC (2014). Differences in the topographic profiles of retinal thickening in eyes with and without serous macular detachment associated with diabetic macular oedema. Br J Ophthalmol..

[13] Turgut B, Gul FC, Ilhan N, Demir T, Celiker U (2010). Comparison of serum glycosylated hemoglobin levels in patients with diabetic cystoid macular edema with and without serous macular detachment. Indian J Ophthalmol..

[14] Ozdemir H, Karacorlu M, Karacorlu SA (2005). Regression of serous macular detachment after intravitreal triamcinolone acetonide in patients with diabetic macular edema. Am J Ophthalmol..

[15] Maalej A, Turki W, Hadj Alouane B, Rannen R (2009). Prognosis factors in diabetic macular edema: am OCT study. J Fr Ophthalmol..

[16] Murakami T, Nishijima K, Sakamoto A, Ota M, Horii T Yoshimura N (2011). Association of pathomorphology, photoreceptor status, and retinal thickness with visual acuity in diabetic retinopathy. Am J Ophthalmol..

[17] Seo KH, Yu SY, Kim M, Kwak HW (2016). Visual and morphologic outcomes of intravitreal ranibizumab for diabetic macular edema based on optical coherence tomography patterns. Retina..

[18] Koytak A, Altinisik M, Sogutoglu Sari E, Artunay O, Akkan JCU, Tuncer K (2013). Effect of a single intravitreal bevacizumab injections on different optical coherence tomographic patterns of diabetic macular oedema. Eye..

[19] Kim M, Lee P, Kim Y, Yu S-Y, Kwak H-W (2011). Effect of intravitreal bevacizumab based on optical coherence tomography patterns of diabetic macular edema. Ophthalmologica..

[20] Yamamoto T, Akabane N, Takeuchi S (2001). Vitrectomy for diabetic macular edema: the role of posterior vitreous detachment and epimacular membrane. Am J Ophthalmol..

[21] Gallemore RP, Jumper JM, McCuen BW, Jaffe GJ, Postel EA, Toth CA (2000). Diagnosis of vitreoretinal adhesions in macular disease with optical coherence tomography. Retina..

[22] Meuer SM, Myers CE, Klein BE, Swift MK, Huang Y, Gangaputra S, Pak JW, Danis RP, Klein R (2015). The epidemiology of vitreoretinal interface abnormalities as detected by spectral-domain optical coherence tomography: the beaver dam eye study. Opthalmology..

[23] Kulikov AN, Sosnovskii SV, Berezin RD, Maltsev DS, Oskanov DZ, Gribanov NA (2017). Vitreoretinal interface abnormalities in diabetic macular edema and effectiveness of anti-VEGF theraphy: an optical coherence tomography study. Clin Ophthalmol..

[24] Lai IA, Hsu WC, Yang CM, Hsieh YT (2017). Prognostic factors of short-term outcomes of intavitreal ranibizumab in diabetic macular edema. Int J Ophthalmol.

[25] Wong Y, Steel DHW, Habib MS, Stubbing-Moore A, Bajwa D, Avery PJ, Sunderland Eye Infirmary study group (2017). Vitreoretinal interface abnormalities in patients treated with ranibizumab for diabetic macular oedema. Graefes Arch Clin Exp Ophthalmol..

[26] Sun J, Lin M, Lammer J, Prager S, Sarangi R, Silva P, Aivello LP (2014). Disorganization of the retinal inner layers as a predictor of visual acuity in eyes with centerinvolved diabetic macular edema. JAMA Ophthalmol..

[27] Nicholson L, Ramu J, Triantafyllopoulou I, Patrao NV, Comyn O, Hykin P, Sivaprasad S (2015). Diagnostic accuracy of disorganization of the retinal inner layers in detecting macular capillary non-perfusion in diabetic retinopathy. Clinical and Experimental Ophthalmology.

[28] Radwan SH, Soliman AZ, Tokarev J, Zhang L, van Kuijk FJ, Koozekanani DD (2015). Association of disorganization of retinal iner layers with vision after resolution of center-involved diabetic macular edema. JAMA Ophthalmol..

[29] Das R, Spence G, Hogg RE, Stevenson M, Chakravarthy U (2018). Disorganization of iner retina and outer retinal morphology in diabetic macular edema. JAMA Ophthalmol..

[30] Tao LW, Wu Z, Guymer RH, Luu CD (2016). Ellipsoid zone on optical coherence tomography: a review. Clin Exp Ophthalmol..

[31] Iacono P, Parodi MB, Scaramuzzi M, Bandello F (2017). Morphological and functional changes in recalcitrant diabetic macular oedema after intravitreal dexamethasone implant. Br J Ophthalmol..

[32] Mori Y, Suzuma K, Uji A, Ishihara K, Yoshitake S, Fujimoto M, Dodo Y, Yoshitake T, Miwa Y, Murakami T (2016). Restoration of foveal photoreceptors after intravitreal ranibizumab injections for diabetic macular edema. Sci Rep..

[33] Santos AR, Costa MA, Schwartz C, Alves D, Figueira J, Silva R, Cunha-Vaz J (2018). Optical coherence tomography baseline predictors for initial best-corrected visual acuity response to intravitreal anti-vascular endothelial growth factor treatment in eyes with diabetic macular edema:The Chartres Study. Retina..

[34] Serizawa S, Ohkoshi K, Minowa Y, Soejima K (2016). Interdigitation zone and band restoration after treatment of diabetic macular edema. Curr Eye Res..

